# Supplementary material for: Hybrid Models and Biological Model Reduction with PyDSTool
Source: PLoS Comput Biol. 2012 Aug 9;8(8):e1002628. doi: 10.1371/journal.pcbi.1002628 (PMC3415397; doi:10.1371/journal.pcbi.1002628)
Supplement: Text S4 — Complete source code for the PyDSTool package (version 0.88.120504). Includes API documentation and help files linking to web pages. This file is identical to the current public release on Sourceforge.net. (ZIP) [file pcbi.1002628.s004.zip › PyDSTool/html/PyDSTool.Toolbox.phaseplane.max_curvature_zone_node-class.html]

xml version="1.0" encoding="ascii"?


PyDSTool.Toolbox.phaseplane.max\_curvature\_zone\_node


| Home | Trees | Indices | Help | PyDSTool |
| --- | --- | --- | --- | --- |

Package PyDSTool ::
Package Toolbox ::
Module phaseplane ::
Class max\_curvature\_zone\_node

[hide private]

[frames] | no frames]

# Class max\_curvature\_zone\_node

source code

```
       object --+                    
                |                    
 MProject.feature --+                
                    |                
MProject.feature_node --+            
                        |            
 MProject.ql_feature_node --+        
                            |        
                    zone_node --+    
                                |    
              nullcline_zone_node --+
                                    |
                                   max_curvature_zone_node
```

---

Find all zones with locally maximal curvature. Optional selection of
those facing up or down only: par: direction (-1, 0, 1, where 0 selects
either (default))


|  |  |
| --- | --- |
| Nested Classes | [hide private] |

\_leaf\_class  
A single zone of locally maximal curvature


|  |  |
| --- | --- |
| Instance Methods | [hide private] |

|  |  |
| --- | --- |
| evaluate(self, nullc)  Default method: evaluate sub-features in order (assumes they are stored as a list). | source code |

**Inherited from `zone_node`**:
`finish`

**Inherited from `zone_node`** (private):
`_local_init`

**Inherited from `MProject.feature_node`**:
`__getitem__`,
`__init__`,
`__repr__`,
`__str__`,
`propagate_verbosity`,
`set_ref_traj`

**Inherited from `MProject.feature_node`** (private):
`_residual_info`

**Inherited from `MProject.feature`**:
`__call__`,
`__eq__`,
`__hash__`,
`__ne__`,
`info`,
`postprocess_ref_traj`,
`prepare`,
`reset_metric`,
`validate`

**Inherited from `MProject.feature`** (private):
`_find_idx`

**Inherited from `object`**:
`__delattr__`,
`__getattribute__`,
`__new__`,
`__reduce__`,
`__reduce_ex__`,
`__setattr__`


|  |  |
| --- | --- |
| Properties | [hide private] |

**Inherited from `object`**:
`__class__`


|  |  |
| --- | --- |
| Method Details | [hide private] |

|  |  |
| --- | --- |
| evaluate(self, nullc) | source code |

Default method: evaluate sub-features in order (assumes they are
stored as a list).

Can override with more sophisticated method (e.g. for use with a
dictionary of sub-features).

Overrides: MProject.feature.evaluate

  


| Home | Trees | Indices | Help | PyDSTool |
| --- | --- | --- | --- | --- |

|  |  |
| --- | --- |
| Generated by Epydoc 3.0.1 on Fri May 4 15:24:09 2012 | http://epydoc.sourceforge.net |
